# Single-dose pharmacokinetics of 2 or 3 tablets of biphasic immediate-release/extended-release hydrocodone bitartrate/acetaminophen (MNK-155) under fed and fasted conditions: two randomized open-label trials

**DOI:** 10.1186/s40360-015-0032-y

**Published:** 2015-11-27

**Authors:** Krishna Devarakonda, Kenneth Kostenbader, Michael J. Giuliani, Jim L. Young

**Affiliations:** Department of Clinical Pharmacology, Mallinckrodt Pharmaceuticals, Hazelwood, MO USA; Independent Pharmaceuticals Professional, Mallinckrodt Pharmaceuticals, Hazelwood, MO USA; Research and Development, Mallinckrodt Pharmaceuticals, Hazelwood, MO USA; Clinical Affairs and Program Management, Mallinckrodt Pharmaceuticals, Hazelwood, MO USA; Department of Clinical Pharmacology, Mallinckrodt Inc., 675 James S. McDonnell Blvd, Hazelwood, MO 63042 USA

**Keywords:** Acetaminophen, Analgesic, Bioavailability, Fasted, Fed, Fixed-dose combination, Hydrocodone bitartrate, Pharmacokinetics

## Abstract

**Background:**

Biphasic immediate-release (IR)/extended-release (ER) hydrocodone bitartrate (HB)/acetaminophen (APAP) 7.5/325-mg tablets are formulated with gastroretentive ER drug delivery technology that has been associated with clinically meaningful food effects in other approved products. Two phase 1 studies evaluated potential effects of food on single-dose pharmacokinetics of IR/ER HB/APAP tablets.

**Methods:**

These were single-center, open-label, randomized, single-dose, 3-period crossover studies in healthy volunteers (aged 18–55 years). IR/ER HB/APAP was administered as a single 2-tablet dose (study 1) or 3-tablet dose (study 2) under fed (high- and low-fat) and fasted conditions. Area under the plasma concentration-time curve from 0 h to time t (AUC_0–t_) and from time 0 extrapolated to infinity (AUC_0–inf_) and maximum observed plasma concentration (C_max_) of hydrocodone and APAP under fed versus fasted conditions were compared using analysis of variance. A 90 % confidence interval of the geometric least squares mean ratio fully contained within 80 to 125 % indicated no treatment difference. Safety and tolerability were assessed.

**Results:**

Forty of 48 participants in study 1 and 21 of 30 in study 2 completed all treatments. In both studies, under fed (high- or low-fat meal) versus fasted conditions, 90 % CIs for AUC_0–t_ and AUC_0–inf_ for both hydrocodone and APAP were entirely contained within the bioequivalent range (80–125 %), indicating that high- and low-fat meals did not affect the extent of exposure. In both studies, a high-fat meal did not affect the C_max_ for hydrocodone. Hydrocodone C_max_ was not affected by a low-fat meal in study 1 but increased by approximately 19 % in study 2. A high-fat meal decreased APAP C_max_ by approximately 20 % (study 1) and 13 % (study 2); a low-fat meal decreased APAP C_max_ by 22 % (study 1) and 21 % (study 2). Approximately 50 % of participants in both studies reported ≥1 treatment-emergent adverse event (TEAE), with no notable difference based on food intake. There were no serious or severe AEs. The most common TEAEs were nausea, vomiting, and dizziness.

**Conclusions:**

Pharmacokinetic and safety findings were similar regardless of food intake. TEAEs were consistent with those reported with low-dose combination opioids. IR/ER HB/APAP can be administered without regard to food.

**Trial registration:**

ClinicalTrials.gov NCT02561650 and NCT02561741.

**Electronic supplementary material:**

The online version of this article (doi:10.1186/s40360-015-0032-y) contains supplementary material, which is available to authorized users.

## Background

Typically, acute pain is treated with immediate-release (IR) opioid formulations to facilitate rapid onset of analgesia, whereas extended-release (ER) formulations are preferred for chronic pain because they provide long-lasting analgesia with less-frequent dosing [1]. Additional potential advantages of ER opioids include fewer concentration peaks and troughs throughout the day, which may improve pain control and compliance, as well as prevent sleep disruption as a result of less-frequent dosing [2, 3]. Fixed-dose combination (FDC) opioid analgesics may provide additive analgesic efficacy while allowing a reduction in the total dose of each component; this may reduce the risk of dose-related adverse events (AEs) associated with either component administered as monotherapy [4, 5]. However, until recently, no opioid FDC with ER properties has been available.

The most frequently prescribed drug (of any kind) in the United States is the FDC analgesic IR hydrocodone bitartrate (HB)/acetaminophen (APAP) [6]. Biphasic IR/ER HB/APAP 7.5/325-mg tablets (MNK-155; Mallinckrodt Pharmaceuticals, Hazelwood, MO) are in development for the treatment of moderate to severe acute pain for which nonopioid analgesics are inadequate. IR/ER HB/APAP delivers 25 % of its HB and 50 % of the APAP from an IR layer for rapid onset of analgesia and employs a gastroretentive matrix (Acuform®, Depomed, Inc., Newark, CA) to allow sustained release of the remainder of the HB (75 %) and APAP (50 %) over a 12-h dosing period. A similarly formulated IR/ER oxycodone/APAP tablet (XARTEMIS™ XR; formerly MNK-795, Mallinckrodt Brand Pharmaceuticals, Inc., Hazelwood, MO) was approved for the management of acute pain severe enough to require opioid treatment and for which alternative treatment options are inadequate [7].

Given that IR/ER HB/APAP is formulated to provide both a rapid and an extended duration of action, it is necessary to characterize the extent and rate of exposure of hydrocodone and APAP during treatment with IR/ER HB/APAP in patients treated under different conditions. In two phase 1 trials, single- and steady-state administration of IR/ER HB/APAP under fasted conditions was found to provide total and peak exposure similar to IR HB/APAP with less-frequent dosing (Data on file, Mallinckrodt, Hazelwood, MO). Potential effects of food on absorption of IR/ER HB/APAP have yet to be characterized.

Clinically relevant food effects have been observed with ER gabapentin tablets (Gralise®, Depomed, Inc., Newark, CA) [8] and ER metformin HCl tablets (Glumetza®, Santarus, Inc., San Diego, CA) [9] formulated using the same gastroretentive matrix used for IR/ER HB/APAP and IR/ER OC/APAP, with high fat content (>50 % calories from fat) augmenting these effects. For ER gabapentin, compared with the fasted state, low-fat and high-fat meals increased the extent of absorption as measured by maximum observed plasma concentration (C_max_) by 33 to 84 %, respectively, and area under the plasma concentration-time curve (AUC) by 33 to 118 %, respectively [8]. For ER metformin HCl, compared with the fasted state, low-fat and high-fat meals increased AUC by 38 and 73 %, respectively, but C_max_ was unaffected. However, for IR/ER OC/APAP, food and fat content do not alter the extent or rate of absorption of oxycodone or APAP compared with the fasted state [10].

It is important to ascertain whether food and fat content of a meal might affect the rate and extent of absorption of the active pharmaceutical ingredients of IR/ER HB/APAP relative to the fasted state, as observed with some products using the same ER matrix. To that end, two phase 1 studies in healthy adult volunteers evaluated the effect of fed (high- and low-fat meals) versus fasted conditions on single-dose pharmacokinetics and bioavailability following administration of IR/ER HB/APAP 7.5/325-mg tablets given as a single 2-tablet dose (study 1) or a single 3-tablet dose (study 2).

## Methods

### Study design

Two phase 1, single-center (PPD Phase I Clinic, Austin, TX, USA), open-label, randomized, single-dose, 3-period crossover trials were conducted. In both studies, participants underwent screening evaluations to determine eligibility within 30 days of period 1 check-in. During the treatment period, participants were randomly assigned to 1 of 6 sequences of 3 treatments, each separated by a 6-day washout interval.

Each study received institutional review board approval (IntegReview Ethical Review Board, Austin, TX, USA) and was conducted in accordance with the Good Clinical Practice Guidelines of the International Conference on Harmonisation. All participants provided written informed consent. The two trials that were registered with ClinicalTrials.gov had the numbers: NCT02561650 and NCT02561741.

### Study population

#### Inclusion criteria

Participants were men or nonpregnant, nonlactating women aged 18 to 55 years with health status considered by investigators as “healthy normal” at screening and check-in assessments. Participants had a body mass index 19 to 30 kg/m^2^ with a minimum weight of 110 lb (women) or 130 lb (men).

#### Exclusion criteria

Exclusion criteria included an acute illness within 14 days before period 1 check-in; electrocardiogram abnormalities; laboratory results falling outside of the upper or lower limits of normal; history of significant psychiatric illness requiring hospitalization, psychotherapy, and/or medication within the previous 3 years. Participants were also excluded if they had a history of any condition that might interfere with the absorption, distribution, metabolism, or excretion of the study drug.

Participants were excluded from the study if, during any study period, they experienced emesis any time after dosing at hour 0 through the 48-h postdose blood collection. This was to ensure that participants had adequate drug exposure to allow accurate assessment of pharmacokinetic parameters. Additional exclusion criteria included positive urine test results for drugs of abuse or alcohol; history of or treatment for substance abuse, narcotic addiction; use of nicotine-containing products within 6 months before period 1 check-in; and use of prescription drugs or nonprescription drugs, vitamins, minerals, or dietary/herbal supplements within 14 days before period 1 check-in and for the duration of the study. Finally, participants were excluded if they had undergone abdominal and/or pelvic surgery, including cholecystectomy, gastric bypass, or gastric band surgery, and cardiothoracic surgery.

### Study treatments

IR/ER HB/APAP 7.5/325-mg tablets were given as a single 2-tablet dose (study 1) or a single 3-tablet dose (study 2) under fasted (reference) and fed (test) conditions. For their predose meals, participants received a US Food and Drug Administration–standardized high-fat meal with approximately 50 % of 1000 ± 100 cal coming from fat [treatment A], a low-fat meal with 25 to 30 % of 800 ± 80 cal coming from fat [treatment B] meal), or fasted (treatment C). To successfully complete the study, participants were required to complete all 3 periods (completers).

### Assessments

#### Blood samples and pharmacokinetics

AUC from 0 h to time t (AUC_0–t_) and from time 0 extrapolated to infinity (AUC_0–inf_) and C_max_ of hydrocodone and APAP were compared across the 3 treatments (high-fat meal, low-fat meal, and fasting). Time to C_max_ (t_max_), time to first measurable concentration (t_lag_), apparent first-order terminal elimination rate constant (K_el_), and apparent plasma terminal elimination half-life (t_1/2_) were also assessed.

For determination of hydrocodone and APAP concentrations, whole blood samples (6 mL) were collected via venipuncture from each participant in prechilled lavender-top vacuum blood collection tubes containing K_2_EDTA anticoagulant. Blood was collected at predose (up to 60 min before dosing); after dosing at 15, 30, and 45 min: and at 1, 2, 3, 4, 5, 6, 7, 8, 9, 10, 11, 12, 16, 18, 20, 24, 36, and 48 h. Blood samples were placed in an ice bath/cryoblock immediately after collection and centrifuged at approximately 4 °C. Within 1 h of collection, the plasma fraction was withdrawn by pipette, divided equally into 2 aliquots in labeled polypropylene screw-cap tubes, and frozen to ≤ −70 °C (study 1) or ≤ −20 °C (study 2). Samples were shipped from the PPD Phase I Clinic and remained frozen until received for assay at the PPD Bioanalytical Lab (Middleton, WI). A summary of the bioanalytical method is listed in an additional file (see Additional file 1). Hydrocodone and APAP concentrations were measured using a high-performance liquid chromatography/tandem mass spectrometry assay that was validated over a calibration range of 0.100 to 50.0 ng/mL for HC and a range of 100 to 50,000 ng/mL (study 1) or 15,000 ng/mL (study 2) for APAP. HC-d6 and APAP-d4 were used as the internal standards. Study data were collected using the Analyst Version 1.4.2 (Applied Biosystems, Carlsbad, CA) and PPD Assist LIMS Version 5 (PPD, Richmond, VA). The assay method was validated for linearity, precision, accuracy, ruggedness, recovery, and specificity. Studies to confirm both short-term stability and long-term storage stability were performed. Analyses of samples were performed following the principles of Good Laboratory Practice and PPD’s standard operating procedures.

#### Safety and tolerability

Treatment-emergent adverse events (TEAEs), serious adverse events (SAEs), vital signs, and pulse oximetry were assessed at baseline and throughout the 48 h after dosing. TEAEs, treatment-related TEAEs, severity of TEAEs, and TEAEs leading to early discontinuation were summarized by system organ class and were coded using the *Medical Dictionary for Regulatory Activities, version 14.0*. Physical examinations were performed at screening, check-in to each study period, and at study exit or early termination. A 12-lead electrocardiogram and laboratory tests (chemistry, hematology, and urinalysis) were performed at screening and final visit or early termination.

### Statistical methods

#### Blood samples and pharmacokinetics

Individual plasma concentration versus time data were used to estimate the pharmacokinetic parameters of hydrocodone and APAP by standard noncompartmental methods. Pharmacokinetic parameters were summarized by treatment using descriptive conditions. Geometric means were included for AUC_0–t_, AUC_0–inf_, and C_max._

To analyze the effect of food, an analysis of variance using the SAS/STAT® version 9.1.3 (SAS Institute, Cary, NC) general linear mixed model procedure was conducted with the natural log-transformed pharmacokinetic parameters (AUC_0–t_, AUC_0–inf_, and C_max_) as the dependent variables, with sequence, treatment, and period as fixed effects and participants nested within sequence as a random effect. Treatment A (fed condition, high-fat meal) was compared to Treatment C (fasted condition), Treatment B (test: fed condition, low-fat meal) was compared to Treatment C (reference: fasted condition), and Treatment A (test: fed condition, high-fat meal) was compared to Treatment B (reference: fed condition, low-fat meal). A 90 % confidence interval (CI) of the geometric least squares mean (LS mean) ratio fully contained within 80.00 to 125.00 % for AUC_0–t_, AUC_0–inf_, and C_max_ indicated no difference between each treatment.

The Wilcoxon signed-rank test was performed to evaluate differences in t_max_ and t_lag_, with a *P* value ≤0.05 indicating a significant difference between treatments.

#### Safety and tolerability

Safety was assessed in all participants who received ≥1 dose of study drug (all dosed participants). All TEAEs were summarized using descriptive statistics.

## Results

### Participant disposition and characteristics

A total of 48 participants were enrolled in study 1, and 30 were enrolled in study 2; 40 participants completed study 1, and 21 completed study 2. Of the 78 participants in total, 15 discontinued because of AEs, of which 14 discontinued because of the TEAE of vomiting, as required by the protocol. Twelve of the 14 participants who vomited were women.

Demographics and baseline characteristics of all dosed participants and completers of both studies are summarized in Table 1. Mean age, height, weight, and body mass index were generally similar between all dosed participants in study 1 and study 2. All participants in study 1 were either white or black; study 2 enrolled 1 participant each of Asian and American Indian/Alaskan Native descent.

**Table 1:** Demographics and baseline characteristics of all dosed participants and completers in studies 1 and 2

Characteristics	Study 1 (2 tablets IR/ER HB/APAP)		Study 2 (3 tablets IR/ER HB/APAP)	
	All dosed participants	Completers	All dosed participants	Completers
	(*N* = 48)	(*n* = 40)	(*N* = 30)	(*n* = 21)
Mean (SD) age, y	33.3 (10.1)	33.2 (10.3)	35.2 (10.8)	36.1 (10.4)
Women, n (%)	24 (50.0)	18 (45.0)	15 (50.0)	7 (33.3)
Race, n (%)				
White	36 (75.0)	31 (77.5)	25 (83.3)	18 (85.7)
Black	12 (25.0)	9 (22.5)	3 (10.0)	1 (4.8)
Asian	0	0	1 (3.3)	1 (4.8)
American Indian or Alaskan Native	0	0	1 (3.3)	1 (4.8)
Ethnicity, n (%)				
Hispanic or Latino	17 (35.4)	15 (37.5)	11 (36.7)	7 (33.3)
Not Hispanic or Latino	31 (64.6)	25 (62.5)	19 (63.3)	14 (66.7)
Mean (SD) height, cm	167.9 (9.3)	168.6 (8.8)	168.7 (9.5)	171.1 (9.7)
Mean (SD) weight, kg	72.6 (12.3)	73.6 (12.6)	74.3 (11.0)	76.7 (11.5)
Mean (SD) body mass index, kg/m^2^	25.6 (3.0)	25.8 (3.1)	26.1 (2.8)	26.1 (2.5)

APAP = acetaminophen; ER = extended release; HB = hydrocodone bitartrate; IR = immediate release

### Pharmacokinetics

#### Hydrocodone

**Fig. 1:**
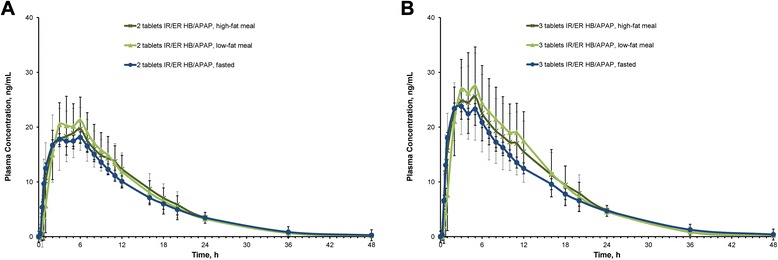
Mean plasma concentration of hydrocodone in (**a**) study 1 and (**b**) study 2

In both studies, plasma hydrocodone concentrations rose rapidly following administration of a single 2-tablet or 3-tablet dose of IR/ER HB/APAP, exhibiting a single peak concentration early in treatment with no trough between the IR and ER phases (Fig. 1). With the 2-tablet dose, plasma concentration peaked at 6 h after a high-fat meal and 4 h after a low-fat meal or under fasted conditions. Peak plasma concentrations with the 3-tablet dose were reached after 4 h under fasted conditions and both fed conditions.

After a single dose of IR/ER HB/APAP, mean total (AUC) exposure and peak (C_max_) exposure to hydrocodone were slightly higher after low-fat and high-fat meals compared with fasted conditions with both the 2-tablet (study 1) and 3-tablet (study 2) doses, but the differences were not clinically meaningful (Table 2). Median t_max_ of hydrocodone was unchanged compared with fasted conditions after a low-fat meal and delayed 2 h after a high-fat meal after the 2-tablet dose compared with the fasted condition. Median t_max_ was delayed by 1 h after the low-fat and high-fat meals after the 3-tablet dose. Median absorption lag time (t_lag_) of hydrocodone increased by 0.25 h when IR/ER HB/APAP was administered after a low-fat meal compared with fasted conditions in both studies.

**Table 2:** Pharmacokinetic estimates under fed and fasted conditions in study completers of studies 1 and 2

Drug/Parameter, geometric mean (SD)	Study 1 (2 tablets IR/ER HB/APAP; *n* = 40^a^)			Study 2 (3 tablets IR/ER HB/APAP; *n* = 21^b^)		
	High-fat meal	Low-fat meal	Fasted	High-fat meal	Low-fat meal	Fasted
Hydrocodone						
AUC_0–t_, ng•h/mL	301.50 (52.81)	299.72 (57.01)	280.10 (58.80)	392.61 (98.89)	401.63 (99.51)	361.21 (83.32)
AUC_0–inf_, ng•h/mL	303.66 (53.13)	301.95 (57.83)	282.94 (59.86)	395.03 (99.82)	404.25 (100.35)	365.70 (85.51)
C_max_, ng/mL	21.66 (4.88)	23.09 (3.79)	20.33 (4.33)	28.04 (6.29)	29.58 (6.69)	25.42 (6.15)
t_max_, h^c^	6.00 (2.00–11.00)	4.00 (2.00–7.00)	4.00 (2.00–7.00)	4.00 (1.00–12.00)	4.00 (2.00–12.00)	3.00 (1.00–5.00)
t_lag_, h^c^	0.00 (0.00–1.07)	0.25 (0.00–0.75)	0.00 (0.00–0.50)	0.00 (0.00–0.75)	0.25 (0.00–0.50)	0.00 (0.00–0.25)
t_½_, h	5.58 (0.85)	5.85 (1.00)	6.43 (1.11)	5.25 (0.85)	5.54 (0.78)	6.35 (1.52)
APAP						
AUC_0–t_, ng•h/mL	33,210.39 (10,402.75)	32,415.11 (9586.52)	32,149.34 (9431.97)	46,656.25 (12,228.35)	47,730.72 (12,238.72)	47,702.26 (13,034.74)
AUC_0–inf_, ng•h/mL	34,689.91 (10,672.37)	34,092.21 (9949.21)	34,803.59 (9635.34)	48,227.95 (12,104.97)	50,010.40 (12,223.51)	51,623.79 (11,493.91)
C_max_, ng/mL	4317.00 (1185.08)	4122.25 (877.19)	5307.00 (1419.43)	6250.95 (1646.83)	5733.33 (1389.04)	7740.48 (2488.56)
t_max_, h^c^	2.00 (0.25–6.05)	2.00 (0.75–7.00)	0.75 (0.25–5.00)	1.00 (0.50–3.00)	3.00 (0.50–8.00)	0.75 (0.50–2.00)
t_lag_, h^c^	0.00 (0.00–0.63)	0.25 (0.00–0.50)	0.00 (0.00–0.25)	0.00 (0.00–0.25)	0.00 (0.00–0.50)	0.00 (0.00–0.25)
t_½_, h	5.37 (2.02)	5.68 (1.68)	7.37 (2.77)	5.80 (1.72)	7.07 (2.53)	8.13 (1.90)

APAP = acetaminophen; AUC_0–inf_ = area under the plasma concentration-time curve from time 0 extrapolated to infinity; AUC_0–t_ = area under the plasma concentration-time curve from 0 h to time t; C_max_ = maximum observed plasma concentration; ER = extended release; HB = hydrocodone bitartrate; IR = immediate release; t_½_ = apparent plasma terminal elimination half-life; t_lag_ = time to first measurable concentration; t_max_ = time to achieve C_max_

^a^With the exception of APAP fasted AUC_0–inf_ (*n* = 38) and APAP fasted t_½_ (*n* = 39)

^b^With the exception of APAP AUC_0–-inf and_ t_½_ for each condition (*n* = 20)

^c^Median (range)

After a single 2- or 3-tablet dose of IR/ER HB/APAP, 90 % CIs for AUC_0–t_ and AUC_0–inf_ for hydrocodone were entirely contained within the bioequivalent range (80–125 % of fasted-state value), indicating that the high-fat and low-fat meals did not affect the extent of exposure. After the 2-tablet dose, 90 % CI for C_max_ for hydrocodone was entirely contained within the bioequivalent range when administered with a high-fat or low-fat meal (Table 3). After the 3-tablet dose, 90 % CIs for C_max_ for hydrocodone were within the bioequivalent range when administered with a high-fat meal but were partially outside the bioequivalent range (110.9–128.3 %) when administered with a low-fat meal, indicating that a low-fat meal increased peak exposure by approximately 19 % compared with the fasted state.

**Table 3:** Geometric LS mean ratios in study completers in studies 1 and 2

Drug/Parameter, % (90 % CI)	Study 1 (2 tablets IR/ER HB/APAP; *n* = 40^a^)			Study 2 (3 tablets IR/ER HB/APAP; *n* = 21^b^)		
	High-fat meal/Fasted	Low-fat meal/Fasted	High-fat meal/Low-fat meal	High-fat meal/Fasted	Low-fat meal/Fasted	High-fat meal/Low-fat meal
Hydrocodone						
AUC_0–t_, ng•h/mL	108.40 (104.44–112.51)	107.45 (103.53–111.52)	100.89 (97.22–104.69)	109.32 (105.53–113.25)	111.63 (107.83–115.55)	97.93 (94.67–101.32)
AUC_0–inf_, ng•h/mL	108.11 (104.14–112.24)	107.17 (103.23–111.26)	100.88 (97.19–104.72)	108.64 (104.87–112.54)	111.01 (107.24–114.92)	97.86 (94.60–101.24)
C_max_, ng/mL	106.66 (100.54–113.15)	114.75 (108.16–121.73)	92.95 (87.64–98.59)	114.71 (106.45–123.60)	119.27 (110.86–128.33)	96.17 (89.50–103.33)
APAP						
AUC_0–t_, ng•h/mL	102.70 (100.05–105.42)	100.74 (98.15–103.41)	101.94 (99.32–104.63)	101.19 (95.65–107.05)	102.36 (96.87–108.16)	98.86 (93.65–104.36)
AUC_0–inf_, ng•h/mL)	100.32 (97.71–103.01)	98.66 (96.09–101.30)	101.69 (99.04–104.40)	98.30 (92.92–103.99)	101.64 (96.22–107.36)	96.72 (91.71–102.00)
C_max_, ng/mL	80.49 (75.44–85.88)	78.10 (73.20–83.33)	103.06 (96.62–109.93)	86.86 (77.33–97.55)	78.68 (70.23–88.16)	110.39 (98.72–123.43)

APAP = acetaminophen; AUC_0–inf_ = area under the plasma concentration-time curve from time 0 extrapolated to infinity; AUC_0–t_ = area under the plasma concentration-time curve from 0 h to time t; C_max_ = maximum observed plasma concentration; ER = extended release; HB = hydrocodone bitartrate; IR = immediate release; LS = least squares

^a^With the exception of APAP fasted AUC_0–inf_ (*n* = 38)

^b^With the exception of APAP AUC_0–inf for_ each condition (*n* = 20)

#### Acetaminophen

**Fig. 2:**
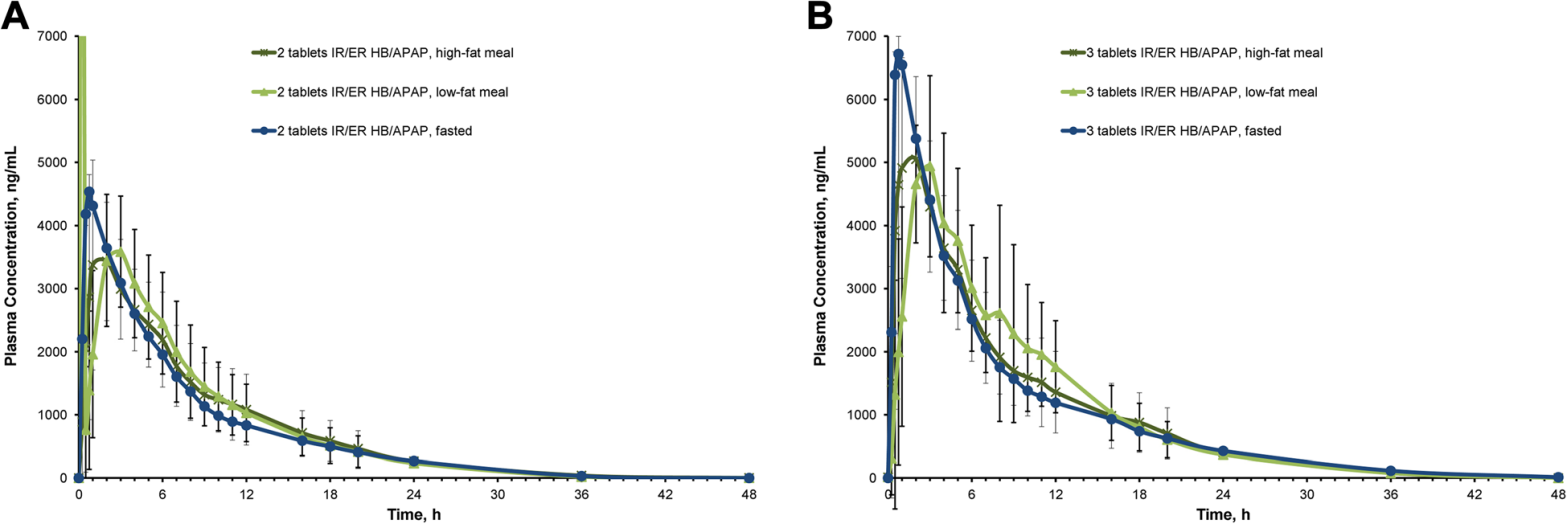
Mean plasma concentration of APAP in (**a**) study 1 and (**b**) study 2. APAP = acetaminophen; ER = extended release; HB = hydrocodone bitartrate; IR = immediate release

Plasma APAP concentrations rapidly increased following administration of a single 2-tablet or 3-tablet dose of IR/ER HB/APAP (Fig. 2). With the 2-tablet dose, C_max_ was attained after 2 h with the high-fat and low-fat meals and <1 h under fasted conditions (Table 2). After 3 tablets, plasma concentrations peaked in 1 h after a high-fat meal, 3 h after a low-fat meal, and in <1 h under fasted conditions.

After 2-tablet and 3-tablet doses, AUC values for APAP were similar under fed and fasted conditions (Table 2). However, with both doses, C_max_ values for APAP were decreased after low-fat and high-fat meals compared with fasted conditions. After a 2-tablet dose, median t_max_ of APAP was delayed by 1.25 h after low-fat and high-fat meals compared with fasted conditions. After the 3-tablet dose, median t_max_ of APAP was delayed by 2.25 h after a low-fat meal but only 0.25 h after a high-fat meal. Median t_lag_ of APAP appeared to be slightly delayed under the low-fat fed condition after the 2-tablet dose but was unaffected after the 3-tablet dose.

In both studies, 90 % CIs for AUC_0–t_ and AUC_0–inf_ for APAP were within the bioequivalent range, indicating that the high- and low-fat meals did not affect the extent of overall exposure (Table 3). After 2-tablet and 3-tablet dosing, 90 % CIs for C_max_ for APAP were partially outside the bioequivalent range compared with the fasted condition, indicating that a high-fat or low-fat meal decreased peak exposure by approximately 20 % with the 2-tablet dose and by 13 and 21 %, respectively, after the 3-tablet dose compared with the fasted condition.

### Safety

Twenty-six (54.2 %) and 15 (50.0 %) participants reported ≥1 TEAE after receiving the 2-tablet (study 1) and 3-tablet (study 2) doses, respectively (Table 4). Although statistical comparisons were not performed, there were no notable differences in the overall incidence of TEAEs after receiving IR/ER HB/APAP under fasted conditions versus with a high-fat or low-fat meal. Individual TEAEs and treatment-related AEs occurred with similar frequencies between the 2-tablet and 3-tablet doses.

**Table 4:** AE incidence and most common TEAEs in all dosed participants in studies 1 and 2

AE, n (%)	Study 1 (2 tablets IR/ER HB/APAP)				Study 2 (3 tablets IR/ER HB/APAP)			
	High-fat meal	Low-fat meal	Fasted	Overall	High-fat meal	Low-fat meal	Fasted	Overall
	*n* = 43	*n* = 42	*n* = 45	*n* = 48	*n* = 24	*n* = 25	*n* = 28	*n* = 30
≥1 TEAE	15 (34.9)	11 (26.2)	15 (33.3)	26 (54.2)	6 (25.0)	6 (24.0)	9 (32.1)	15 (50.0)
≥1 SAE	0	0	0	0	0	0	0	0
≥1 severe TEAE	0	0	0	0	0	0	0	0
Treatment-related TEAEs	13 (30.2)	10 (23.8)	13 (28.9)	24 (50.0)	6 (25.0)	5 (20.0)	9 (32.1)	15 (50.0)
TEAEs leading to study discontinuation	2 (4.7)	2 (4.8)	3 (6.7)	7 (14.6)	3 (12.5)	1 (4.0)	4 (14.3)	8 (26.7)
Most common TEAEs^a^								
Nausea	4 (9.3)	3 (7.1)	7 (15.6)	12 (25.0)	4 (16.7)	3 (12.0)	6 (21.4)	11 (36.7)
Vomiting	2 (4.7)	2 (4.8)	2 (4.4)	6 (12.5)	3 (12.5)	1 (4.0)	4 (14.3)	8 (26.7)
Dizziness	3 (7.0)	4 (9.5)	7 (15.6)	10 (20.8)	3 (12.5)	1 (4.0)	4 (14.3)	6 (20.0)
Headache	4 (9.3)	2 (4.8)	2 (4.4)	6 (12.5)	2 (8.3)	0	0	2 (6.7)
Feeling hot	0	0	1 (2.2)	1 (2.1)	0	1 (4.0)	2 (7.1)	3 (10.0)
Pruritus	1 (2.3)	0	1 (2.2)	2 (4.2)	2 (8.3)	0	1 (3.6)	3 (10.0)

AE = adverse event; APAP = acetaminophen; ER = extended release; HB = hydrocodone bitartrate; IR = immediate release; SAE = serious adverse event; TEAE = treatment-emergent adverse event

^a^Affecting ≥10 % of participants in the overall group in either study

However, discontinuations due to AEs were more frequent with the 3-tablet dose (*n* = 8, 26.7 %) than with the 2-tablet dose (*n* = 7, 14.6 %). This difference was due primarily to a greater frequency of nausea and vomiting with the 3-tablet dose. Vomiting led to study discontinuation in 6 (12.5 %) participants in study 1 and 8 (26.7 %) participants in study 2; one participant discontinued study 1 because of pruritus. Twelve of the 14 participants who vomited were women. The AE of vomiting occurred in 6 participants under fasted conditions and in 3 and 5 participants after a low-and high-fat meal, respectively. Five of 6 participants who vomited under fasted conditions and all 3 who vomited after a low-fat meal did so after receiving their first dose of study medication. In contrast, only 1 of 5 who vomited after a high-fat meal did so after their first dose. In the remaining 5, the dose taken was the second or third dose. The participant who discontinued after receiving the 2-tablet dose because of moderate pruritus was treated with a single 25-mg dose of diphenhydramine, after which the AE resolved.

The most commonly reported individual TEAEs (affecting ≥10 % of participants in the overall group) included nausea, vomiting, and dizziness in both studies. In addition, headache after the 2-tablet dose and pruritus and feeling hot after the 3-tablet dose were reported in ≥10 % of participants. After either dose, all TEAEs were mild to moderate in severity. No severe or serious TEAEs or deaths were reported.

No clinically meaningful changes in clinical laboratory values, vital signs, or mean or individual oxygen saturation values were reported. No 12-lead electrocardiogram abnormalities were noted, and no abnormal physical examination result was considered a TEAE.

## Discussion

Data from two phase 1 studies demonstrated that there is only a minimal food effect with IR/ER HB/APAP 7.5/325-mg tablets, not likely to be of clinical significance. A high-fat meal did not have an effect on hydrocodone total exposure or peak exposure after either a 2-tablet or 3-tablet dose. A low-fat meal did not affect total hydrocodone exposure after a 2-tablet or 3-tablet dose but did increase peak exposure after a 3-tablet dose by approximately 19 %. No effect of food on C_max_ was noted after the 2-tablet dose. These results are consistent with food effects reported for other ER hydrocodone products, where food, particularly a fatty meal, may produce modest increases in hydrocodone C_max_ but have no significant effect on total absorption or require administration on an empty stomach [11, 12]. After either IR/ER HB/APAP dose, hydrocodone was rapidly absorbed under all conditions, with no clinically relevant lag in the appearance of plasma concentrations when administered with food. There was a single peak with no trough between the IR and ER phases, suggesting that the objective of rapid onset of effect with sustained activity thereafter has been achieved.

A high-fat meal decreased APAP C_max_ by approximately 20 % after a 2-tablet dose and 13 % after a 3-tablet dose compared with fasted conditions. A low-fat meal did not have an effect on total APAP exposure with either dose. However, a low-fat meal decreased peak exposure of APAP by approximately 20 % after the 2-tablet dose and 21 % after the 3-tablet dose compared with fasted conditions. Because food had no effects on total exposure, these modest decreases in peak exposure are not expected to be clinically significant or necessitate dosage adjustments. After either dose, APAP was rapidly absorbed under all conditions, with no clinically relevant lag in the appearance of plasma concentrations when administered with food.

Differences in pharmacokinetic results after a 2-tablet dose (study 1) compared with the 3-tablet dose (study 2) were apparent, although inferences about the relative properties of the 2 doses are based on numerical differences without formal statistical analysis. For hydrocodone, mean AUC_0–t_, AUC_0–inf_, and C_max_ were larger in participants who received 3 tablets regardless of fed state compared with participants who received 2 tablets. Under all conditions, mean t_½_ was slightly longer in participants who received 2 tablets compared with those who received 3 tablets. For APAP, mean AUC_0–t_, AUC_0–inf_, and C_max_ were larger in participants who received 3 tablets regardless of fed state compared with participants who received 2 tablets. When comparing each fed state (high-fat, low-fat, and fasting), mean t_½_ was slightly longer in participants who received 3 tablets compared with those who received 2 tablets. Thus, hydrocodone and APAP exposure increased in a predictable manner when the dose increased under all conditions, regardless of food intake.

In the present analysis, following a single dose of IR/ER HB/APAP 7.5/325 mg 2 tablets, nausea was reported in 25 % of participants and vomiting in 13 %; following a single dose of IR/ER HB/APAP 7.5/325 mg 3 tablets, nausea was reported in 37 % of participants and vomiting in 27 %. No severe or serious TEAEs were reported under any treatment condition, and there were no clinically meaningful abnormalities of laboratory values, changes in vital signs, oxygen saturation, electrocardiogram results, or physical examination findings. No notable safety differences were observed between the fed (high-fat and low-fat meals) and fasted treatment groups in either study, with the exception of the TEAEs of nausea and vomiting. Almost all participants who vomited were women, which is consistent with research showing that women are more susceptible to vomiting during opioid therapy [13]. Reasons for this disparity between men and women are not clear. However, there are data suggesting that hormonal factors, differences in sensitivity to opioid agonism, and other factors may be involved [14].

The safety profile of single, 2-tablet and 3-tablet doses of IR/ER HB/APAP was consistent with expectations for a low-dose combination opioid analgesic. In a randomized, double-blind, placebo- and active-controlled study, 63 patients who had undergone impacted molar extraction received a single dose of IR HB/APAP 7.5/500 mg, with ibuprofen (dosage not specified) available as rescue medication; during 6 h of follow-up, nausea and vomiting were reported in 16 and 8 % of patients, respectively [15]. Short-term multi-dose studies of IR HB/APAP at doses of 5/325 mg and 7.5/650 mg and IR HB/ibuprofen 7.5/200 mg 1–2 tablets have reported nausea in 21 to 36 % and vomiting in 4 to 13 % of patients, respectively [16–18].

A limitation of this analysis is that elderly, pediatric, and substantially over- or underweight patients were not represented. Patients with significant illnesses that might affect pharmacokinetics or tolerability were also excluded, and demographic diversity was limited, with only 1 participant each of Asian and American Indian/Alaskan Native descent participating in study 2.

## Conclusions

Food intake had no effect on overall exposure to hydrocodone or APAP with IR/ER HB/APAP. Small changes in C_max_ for both hydrocodone and APAP were observed under fed versus fasted conditions; however, these differences were not considered clinically meaningful. IR/ER HB/APAP was generally well tolerated, with a TEAE profile typical of low-dose opioid combination analgesics, and there was no indication that food intake had any effect on safety. These results suggest that IR/ER HB/APAP can be administered without regard to food.

## Additional file

Additional file 1:
**Summary of the bioanalytical method for determination of hydrocodone and acetaminophen concentrations.** (DOC 52 kb)

## Abbreviations

AE: adverse event
APAP: acetaminophen
AUC: area under the concentration-time curve
AUC_0–t_: AUC from 0 h to time t
AUC_0–inf_: AUC from time 0 extrapolated to infinity
C_max_: maximum plasma concentration
ER: Extended release
HB: hydrocodone bitartrate
IR: immediate release
SAE: serious adverse event
TEAE: treatment-emergent adverse event
t_lag_: time to first measurable concentration
t_max_: time to maximum plasma concentration
t_1/2_: apparent plasma terminal elimination half-life
