# Supplementary material for: Single-dose pharmacokinetics of 2 or 3 tablets of biphasic immediate-release/extended-release hydrocodone bitartrate/acetaminophen (MNK-155) under fed and fasted conditions: two randomized open-label trials
Source: BMC Pharmacol Toxicol. 2015 Nov 27;16:31. doi: 10.1186/s40360-015-0032-y (PMC4662814; doi:10.1186/s40360-015-0032-y)
Supplement: Additional file 1: — Summary of the bioanalytical method for determination of hydrocodone and acetaminophen concentrations. (DOC 52 kb) [file 40360_2015_32_MOESM1_ESM.doc]

**Summary of the bioanalytical method for determination of hydrocodone and acetaminophen concentrations**

| **Parameter** | **Hydrocodone** | | **Acetaminophen** | |
| --- | --- | --- | --- | --- |
|  | **Study 1** | **Study 2** | **Study 1** | **Study 2** |
| Internal standard | Hydrocodone-d_6_ | Hydrocodone-d_6_ | Acetaminophen-d_4_ | Acetaminophen-d_4_ |
| Limit of quantitation, ng/mL | 0.100 | 0.100 | 100 | 100 |
| Average recovery of drug, % | 90.3 | 98.0 | 81.3 | 78.5 |
| Average recovery of internal standard, % | 88.3 | 99.5 | 79.3 | 74.0 |
| Standard curve concentrations, ng/mL | 0.100  0.200  0.360  1.20  4.50  15.0  40.0  50.0 | 0.100  0.200  0.360  1.20  4.50  15.0  40.0  50.0 | 100  200  360  1200  4500  15,000  40,000  50,000 | 100  150  250  750  2000  5000  13,000  15,000 |
| Quality control concentration, ng/mL | 0.100  0.250  0.600  2.40  8.00  37.5 | 0.100  0.250  0.600  2.40  8.00  37.5 | 100  250  600  2400  8000  37,500 | 100  200  450  1200  3500  12,000 |
| Quality Control Intraday precision range, % | 3.00 to 11.5 | 1.38 to 17.3 | 2.81 to 5.82 | 1.86 to 12.3 |
| Quality Control Interday accuracy range, % | –4.11 to 13.7 | 1.25 to 17.8 | –12.3 to 3.16 | –10.5 to 11.1 |
| Quality Control Interday precision range, % | 3.73 to 7.53 | 2.34 to 10.7 | 3.52 to 9.34 | 3.93 to 11.2 |
| Quality Control Intraday accuracy range, % | –0.934 to 6.02 | 4.06 to 16.5 | –6.48 to –0.994 | –0.0845 to 2.39 |
| Bench-top stability, h  (room temperature) | 27.5 | 24.75 | 27.5 | 24.75 |
| Freeze-thaw stability, cycles | 4 cycles at –20°C and  5 cycles at –70°C in the presence of 50.0 ng/mL naltrexone | 5 cycles at –20°C and  5 cycles at –70°C in the presence of 50.0 ng/mL naltrexone | 5 cycles at –20°C and  5 cycles at –70°C in the presence of 50.0 ng/mL naltrexone | Not rep5 cycles at –20°C and  5 cycles at –70°C in the presence of 50.0 ng/mL naltrexone orted |
| Long-term storage stability, d | 309 days at –20°C and  –70°C in K_2_EDTA in human plasma  in the presence of 50.0 ng/mL naltrexone | 216 days at –20°C in K_2_EDTA in human plasma in the presence of 50.0 ng/mL naltrexone | 309 days at –20°C and  –70°C in K_2_EDTA in human plasma  in the presence of 50.0 ng/mL naltrexone | 216 days at –20°C in K_2_EDTA in human plasma in the presence of 50.0 ng/mL naltrexone |
